# Enhanced Control Efficacy of Different Insecticides Mixed with Mineral Oil Against Asian Citrus Psyllid, *Diaphorina citri* Kuwayama, Under Varying Climates

**DOI:** 10.3390/insects16010028

**Published:** 2024-12-30

**Authors:** Wei Hu, Kejing Wang, Xiaoyue Zhong, Pei Jiang, Shunchang Zhang, Zhanjun Lu, Zhixiang Zhang, Long Yi, Ning Zhang

**Affiliations:** 1 National Navel Orange Engineering Research Center, College of Life Sciences, Gannan Normal University, Ganzhou 341000, China; weihu2919@163.com (W.H.); 18166067961@163.com (K.W.); y2727846621@163.com (X.Z.); luzhanjun7@139.com (Z.L.); 2 National Agro-Tech Extension and Service Center, Beijing 100125, China; jiangp2006@126.com; 3 Zhejiang Province Taizhou City Agricultural Means of Production Co., Ltd., Taizhou 318000, China; zsc@tznz.net; 4 National Key Laboratory of Green Pesticide, South China Agricultural University, Guangzhou 510642, China; zdsys@scau.edu.cn; 5 Jiangxi Provincial Key Laboratory of Pest and Disease Control of Featured Horticultural Plants, Gannan Normal University, Ganzhou 341000, China

**Keywords:** *Diaphorina citri*, synthetic insecticides, mineral oil, control efficacy, rainfall

## Abstract

Asian citrus psyllid (ACP), *Diaphorina citri* (Hemiptera: Liviidae), is one of the most devastating pests in citrus orchards due to its role in transmitting Huanglongbing (HLB). Currently, chemical control remains the most effective strategy for ACP management. Mineral oils are commonly used as insecticides or adjuvants in integrated pest management (IPM) practices. To extend the product life of synthetic pesticides, we evaluated the toxicity of chlorpyrifos (CPF), thiamethoxam (THX), or pyriproxyfen (PPF) mixed with mineral oil Lvying (LY) against ACP nymphs under different weather conditions. Individual application of CPF, THX, and PPF effectively controlled against ACP nymphs under no rain conditions, with mortality rates varying from 20 to 100% during 1–5 d after treatment. The addition of LY at 1.0% or 0.5% rates to CPF, THX, and PPF significantly enhanced their control efficacy, achieving sustained mortality rates from 60 to 100% during the same period. Light rain had less impact on the control efficacy of these insecticide mixtures compared to individual insecticides. However, cumulative rainfall above 20 mm significantly reduced the control efficacy of individual insecticides and their mixtures. The addition of LY decreased both surface tension and contact angle of THX solution on citrus leaves, thereby enhancing the solution’s wetting and increasing THX deposition. Moreover, the rainfastness of THX was improved after adding LY, leading to a greater retention on the leaves. LY at a rate of 1.0% exhibited excellent efficacy against ACP nymphs, and observations using scanning electron microscopy (SEM) showed that LY altered ACP mouthpart morphology and blocked spiracles, likely contributing to its insecticidal effects. This study revealed that mineral oils can serve as both insecticides to combine with synthetic pesticides for enhancing toxicity against ACP and as adjuvants to facilitate the deposition and rainfastness of synthetic pesticides on leaves, which could be recommended for sustainable ACP management in citrus orchards.

## Simple Summary

Asian citrus psyllid (*Diaphorina citri*) is an important pest in citrus worldwide because it is the primary vector of *Candidatus* Liberibacter asiaticus, causing Citrus Huanglongbing. Chemical pesticides can effectively control the pest, while the development of insecticide resistance can significantly reduce efficacy of many pesticides or limit their application. The objective of this study was to evaluate the control efficacies of chlorpyrifos, thiamethoxam, or pyriproxyfen mixed with mineral oil Lvying against *D. citri* nymphs. We found all insecticides exhibited enhanced efficacy when mixed with Lvying, with thiamethoxam mixture showing the best efficacy. Lvying facilitated the deposition of thiamethoxam on citrus leaves and also increased its rainfastness. This study offers insights for improving *D. citri* control, enhancing pesticide efficacy, and managing insecticide resistance.

## 1. Introduction

Citrus (*Citrus reticulata* Blanco) is one of the most important fruits in the world, highly favored by consumers for its distinctive flavor profile and rich nutritional composition, including functional polysaccharides, β-carotene, vitamin C, and flavonoids [1]. Therefore, citrus fruits are extensively cultivated globally, with an annual production of approximately 146 million tons, including 38 million tons in China [2,3]. However, over the past decade, citrus production has sharply decreased because of huanglongbing (HLB). HLB is considered the most destructive disease in citrus industry that the causal agent, *Candidatus Liberibacter* asiaticus (*C*Las) [4], disrupts nutrients transport or uptake from the leaves to the roots, leading to symptoms such as chlorosis, stunted growth, sparse foliage, abnormal flowering, dieback of twigs, premature fruit drop, and production of small and misshapen fruits in infected trees [5]. HLB was first found in Florida in 2005 [6], and has caused over USD 3.6 billion of loss in the five years since [7]. Currently, the disease can be found in some of the world’s most prominent citrus producing regions, including the Americas, Indonesia, Brazil, Philippines, South Africa, and China [4,8,9]. There have presently been no effective strategies for the direct control of HLB because the pathogen cannot be cultured artificially. HLB is transmitted by the Asian citrus psyllid (ACP), *Diaphorina citri* Kuwayama [4]. ACP feeds on all commercial citrus species and cultivars, including rootstocks, and the infected citrus hosts reached to 88–100% when ACP fed for 30 min or longer [10]. Therefore, reduction in ACP populations in citrus orchards is a key management approach to prevent the spread of HLB.

Chemical pesticides are mostly used by growers to control psyllid populations in the field, which can rapidly kill the insect vector and then effectively reduce the incidence or spread of HLB. Afidopyropen is considered to have high contact toxicity for the laboratory and field populations of *D. citri* adults; foliar application positively affected the settling of adults on potted ‘Swingle’ citrumelo host, and also higher concentrations reduced the adult fecundity and increased egg mortality [11]. Flupyradifurone exhibited several negative effects to all life stages of *D. citri*, such as inhibiting feeding and settling behavior, initiating flight, as well as suppressing fecundity [12]. Foliar applications of pyrethroid insecticides, including fenpropathrin, lamda-cyhalothrin, and zeta-cypermethrin, as well as organophosphate, such as chlorprifos, dimethoate, tolfenpyrad, phosmet, sulfoxaflor, and methidathion, significantly suppressed nymphs and adults by more than 90% [13]. Bifenthrin and dimethoate could rapidly kill ACP adults under the greenhouse and field conditions, while the control efficacy was shorter in young shoots than in mature shoots because of frequent flushing of young trees [14]. Farmanullah et al. [15] reported that ɑ-cyhalothrin, endosulfan, and methidathion by foliar spraying reduced ACP populations on tender shoots of *Citrus aurantium* at an orchard, while the control efficacy was lower than thiamethoxam. Thiamethoxam belongs to a second-generation systemic neonicotinoid insecticide, which acts as nicotinic acetylcholine receptor agonist [16]. The insecticide is registered in more than 130 countries for foliar, soil, or seed treatment, and is extensively used to control sucking pests, including aphids, whiteflies, and pysllids [16,17]. Tang et al. [18] reported that thiamethoxam exhibited high toxicity against ACP populations, providing effective protection for 30 d under field conditions. Byrne et al. [19] found that thiamethoxam exhibited higher insecticidal activity against ACP than imidacloprid and dinotefuran. Furthermore, insect growth regulators (IGRs) with different mode of actions that disrupt the molting process or cuticle formation in insects are effective alternatives for ACP management. For example, pyriproxyfen, buprofezin, and diflubenzuron were toxic to various developmental stages of *D. citri*, such as inhibiting egg production and egg hatch, inducing nymphal mortality, as well as suppressing adult emergence [20,21].

Effective control of ACP populations requires multiple applications (8–18 per year) of foliar insecticides belonging to pyrethroid, organophosphate, or neonicotinoid classes [22]. However, repeated use of the same insecticide or those with limited modes of action has led to the development of varying levels of resistance in ACP populations [23]. To delay the resistance development and promote sustainable management of ACP, insecticides with different mode of actions are alternately applied or combined with other insecticides having distinct mode of actions. Studies show that mixtures of beta-cypermethrin + thiamethoxam and spirotetramat + bifenthrin achieved rapid and long-term control effects against ACP [24,25]. Additionally, beta-cyfluthrin mixed with thiamethoxam or tolfenpyrad exhibited increased control efficacy and offered extended protection lasting between 3 and 30 d after spraying [26]. Moreover, foliar application of blends containing thiamethoxam and spirotetramat displayed synergistic or additive effects against ACP [18]. Mineral oils, composed of saturated hydrocarbons derived from petroleum with varying chain lengths, are commonly used as pesticides in integrated pest management (IPM) programs to target a broad range of insect pests, such as bean thrips (*Megalurothrips distalis*) [27], aphids (*Myzus persicae*) [28], citrus leafminer (*Phyllocnistis citrella*) [29], citrus red mite (*Panonychus citri*), and red scale (*Aonidiella aurantii*) [30]. Mineral oils are also effective for ACP control, which are not only toxic to adult and nymphs, but also suggest ovicidal effects, emergence inhibition and repellent activity [31,32,33]. Rae et al. [34] showed insecticidal activities of mineral oils on ACP were comparable to those for omethoate and diflubenzuron. Mineral oils are frequently mixed with synthetic insecticides as insecticides or adjuvants to enhance the effectiveness of the active agents, such as imidacloprid [28] and thiacloprid [35].

The control efficacies of pesticides against insect pests are influenced by many factors, such as weather conditions, tree size, growing season, and pest susceptibility degree under field conditions [26]. Foliar application of contact and systemic insecticides in mature citrus orchards achieve better efficacy than in young citrus orchards [14,36]. The organophosphate insecticides chlorpyrifos and dimethoate exhibited higher toxicity to ACP at higher temperature, whereas the pyrethroids fenpropathrin and lambda–cyhalothrin showed a negative temperature-dependent toxicity correlation [37]. Rainfall, in particular, can significantly reduce pesticide retention on plant surfaces [38]. In this study, chlorprifos, thiamethoxam, and pyriproxyfen, representing organophosphate, neonicotinoid, and IGRs insecticides, respectively, were selected to evaluate the toxicity against ACP nymphs when mixed with a mineral oil (Lvying) under varying weather conditions. We also investigated the mechanisms underlying the enhanced efficacy, including their effects on pesticide deposition, rainfastness, and ACP morphology. Our work contributes to extending the life of synthetic pesticides by adding mineral oils and provides valuable information for improving the effectiveness of ACP control and to implement integrated pest management strategies to delay insecticide resistance.

## 2. Materials and Methods

### 2.1. Insects

Asian citrus psyllids were collected from an orange grove in June 2014 and maintained in a greenhouse at National Navel Orange Engineering Research Center, Gannan Normal University, Ganzhou, China. The insect population was reared on *Murraya exotica* at 26 ± 1 °C, 70% ± 5% relative humidity (RH) and a 14:10 h (light:dark) photoperiod for more than 80 generations without exposure to pesticides before the experiment.

### 2.2. Chemicals

Lvying (LY, 99% mineral oil) was obtained from Hanyu SK ETS Co., Ltd. (Ulsan, Republic of Korea). Chlorpyrifos (CPF, 45% emulsifiable concentrate), thiamethoxam (THX, 30% suspension concentrate), and pyriproxyfen (PPF, 10% emulsifiable concentrate) were obtained from Shandong Bainongsida Biological Technology Co., Ltd. (Qingzhou, China), Shandong Hailier Chemical Co., Ltd. (Weifang, China), and Guangdong Lvyin Biological Technology Co., Ltd. (Maoming, China), respectively.

Commercial formulations of CPF, THX, and PPF were dissolved in water at a recommended rate of 0.125%, 0.025%, and 0.1% (*v*/*v*), respectively. LY was diluted with water to rates of 1.0%, 0.5%, or 0.2% (*v*/*v*). Each insecticide was mixed with LY at different rates, and the resulting mixtures was denoted as Insecticide&1.0%LY, Insecticide&0.5%LY, and Insecticide&0.2%LY, respectively.

### 2.3. Field Experimental Design

Field trials were conducted in a block of 6-year-old ‘Newhall’ navel oranges on *Potassium trifoliate* rootstocks in Ganzhou from April to June 2022. The experimental design was a randomized complete block with 15 treatments and an untreated control. The 15 treatments included 3 rates of LY, individual applications of CPF, THX, and PPF, and mixtures of each insecticide (CPF, THX, and PPF) with LY at 3 distinct rates. Each treatment consisted of 9 trees divided into 3 plots of three 3 each, separated by 2 buffer rows.

To evaluate the effectiveness of the mixture solutions for ACP control and the impact of rainfall, the pesticides were sprayed on ‘Newhall’ trees until runoff using a handheld sprayer (3WBD-20A, Taizhou Luqiao the Ming Hui Electric Sprayer Co., Taizhou, China) in three separate blocks on April 3, April 24, and June 10. The corresponding weather conditions following each application are indicated in Table 1. The control trees were sprayed with water only. Leaves (40–50) from different directions of each tree were collected at 0.5, 1, 2, 3, 5, 7, and 9 d post-application, and the collected samples for the pesticide residue analyses were stored at −20 °C.

### 2.4. Toxicity Assays

Collected leaves were placed in 90 mm Petri dishes with moistened filter papers to prevent leaf drying. Ten fifth-instar nymphs were transferred onto each leaf using a camel-hair brush. Dishes were maintained in an illumination incubator at 25 °C and 70% RH, with a 14:10 h (light:dark) photoperiod. Nymphs were considered dead when no movement was observed by touching with a camel-hair brush, and the dead number was recorded at 24 h after exposure to the leaves. Each treatment was replicated three times.

### 2.5. Residue Detection of THX

Based on superior efficacy of THX, both individually and in mixtures, we focused on THX residue in citrus leaves treated by THX alone, THX&1.0%LY, THX&0.5%LY, and THX&0.2%LY. The residue was determined as described by Hu et al. [12]. Briefly, 2 g of powdered leaf sample was placed into a 50 mL centrifuge tube, followed by the addition of 2 mL of deionized water to hydrate the matrix. Then, 10 mL of acetonitrile was added and sonicated for 30 min to extract the insecticide. Anhydrous magnesium sulfate (2 g) and sodium chloride (0.5 g) were added into the mixture and immediately vortexed vigorously for 2 min. After centrifugation at 5000 rpm for 5 min, 1.5 mL of the supernatant was transferred to a 15 mL tube containing a mixture of cleanup sorbents (50 mg of primary secondary amine, 150 mg of anhydrous magnesium sulfate and 10 mg of C18), followed by vigorous vortex and centrifugation at 5000 rpm for 5 min. Finally, the supernatant was collected and filtered through 0.22 μm organic-phase membrane filters.

THX content was analyzed using performance liquid chromatography (HPLC) (Agilent Technologies Inc., Santa Clara, California, USA) with a UV detector (Agilent 1260) and an Eclipse XDB-C18 column (4.6 × 250 mm, 5 μm, Agilent). The mobile phase consisted of water, methanol, and acetonitrile (75:15:15, *v*/*v*/*v*) at the flow rate of 1 mL/min. The detection wavelength was set to 254 nm.

### 2.6. Adhesion Properties Observation

THX solutions, including THX alone and THX&1.0%LY, were sprayed onto three-year-old potted citrus trees until to runoff using a small hand sprayer (Linhai Nanye Household Co., Ltd., Linhai, China). After air-drying, the trees were subjected to rainfall using a simulated rainfall system (Shandong Renke Control Technology Co., Ltd., Jinan, China). The simulation was conducted at a light rainfall intensity of 5 mm/h, with a 15 min period of each time repeated 1, 2, 3, and 4 times. Between consecutive simulations, a 2 h interval was maintained to ensure air-drying of the leaves. Citrus leaves from each treatment were collected, dried at 45 °C for 4 h, and THX particles were observed under scanning electron microscopy (SEM) (EVO MA 15, Carl Zeiss Co., Ltd., Oberkochen, Germany).

### 2.7. Characterization of Droplet Surface Tension, Contact Angle and Spreading

Surface tension and contact angles of individual THX solution at a rate of 0.025% and THX&1.0%LY solutions were measured using an optical contact angle meter (Dongguan Shengding Precision Instrument Co., Ltd., Guangdong, China). The droplets (1 μL) of THX and THX&1.0%LY solutions were dripped onto fresh citrus leaves, respectively. The surface tension was measured using an optical contact angle meter, and the contact angle was recorded at intervals 10 s until 210 s. Each treatment was replicated three times. The spreading behavior of each solution was observed on three citrus leaves under an optical microscope (Chongqing Optec Instrument Co., Ltd., Chongqing, China), with 2 μL different droplets for 20 min, taking a picture every 5 min.

### 2.8. Ultrastructure Observation of ACP

Six citrus leaves were soaked in 1.0% LY solution or distilled water (control) for 10 s, and then dried in air. Ten fifth-instar nymphs were put on a treated leaf with a camel hair brush. After exposure for 24 h, live nymphs were collected and fixed in 0.25% glutaraldehyde overnight. Subsequently, the samples were dehydrated with a series of increasing concentrations of ethanol, critical point dried, and coated with gold. The morphology of mouthparts and spiracle of ACP was viewed under SEM. The 1.0% LY treatment was replicated three times.

### 2.9. Statistical Analysis

Data were statistically analyzed using SPSS 26 (SPSS Inc., Chicago, IL, USA), and are presented as means ± standard error of triplicate measurements. One-way analysis of variance (ANOVA) followed by the least significant difference (LSD) test was used to compare significant differences (*p* < 0.05).

## 3. Results

### 3.1. Effects of Different Weather Conditions on Toxicity of the Pesticides

The was found to be enhanced control efficacy of the insecticides mixed with LY under no rain days.

The corresponding mortality rates are shown in Table 2. For LY mineral oil, both 1.0% and 0.5% LY exhibited significant toxicity against ACP nymphs. The 1.0% LY treatment showed increased mortality from 83% at 0.5 d to peaking at 93% at 2 d, followed by a gradual decline to 67% at 5 d. The efficacy of 0.5% LY gradually decreased from 90% at 0.5 d to 37% at 3 d. The 0.2% LY showed limited practical value for ACP control throughout the experimental period, with mortality rates of 0–30%.

LY mineral oil at higher concentrations of 1.0% and 0.5% significantly enhanced the insecticidal efficacy of the insecticides of CPF, PPF, and THX against ACP, with the exception of 0.5 d, and extended their protective duration, while 0.2% LY had limited improvement in the insecticide effectiveness. For CPF insecticides, individual CP and CPF&0.2%LY exhibited similar efficacy for ACP control, with the mortality rates decreasing substantially from around 80% at 0.5 d to approximately 50% at 2 d. However, the CPF&1.0%LY treatment maintained notably high mortality rates, exceeding 80% throughout the first 5 days of treatment. The CPF&0.5%LY treatment also showed enhanced efficacy compared to CPF alone, although it was slightly less effective than CPF&1.0%LY, maintaining 67% mortality at 5 days post-treatment. Similarly, 1.0% and 0.5% LY significantly improved the insecticidal activity of PPF against ACP. The mortality rates in PPF&1.0%LY and PPF&0.5%LY treatments peaked at 1 d post-application, with both treatments exhibiting excellent insecticidal activity against ACP for up to 5 d. In contrast, the efficacy of PPF alone and PPF&0.2%LY sharply decreased after 1 d, with no significant differences between the both treatments.

Among the three insecticides, THX showed the highest control efficacy, maintaining higher mortality rates (>80%) through day 5, particularly when mixed with 1.0% and 0.5% LY. At the first 2 d post-application, THX&1.0%LY and THX&0.5%LY achieved completed mortality (100%). The enhanced efficacy persisted even at 7 d post-application, with 73% mortality rates in both THX&1.0%LY and THX&0.5%LY treatments and 63% in THX&0.2%LY treatment.

### 3.2. Effects of Rainfall on the Toxicity of the Pesticides

The insecticides CPF, PPF, and THX, as well as LY mineral oil, both individually and in mixtures, were sprayed on a rain-free day followed by light rainfall beginning 1 d after application. As shown in Table 3, the control efficacy of all treatments against ACP nymphs at 0.5 d post-application was comparable to that observed on no rain days (Table 2). However, the continuous light rain subsequently resulted in a decreased in pesticide toxicity against ACP nymphs. The 1.0% and 0.5% LY treatments showed a rapid decline in efficacy. The mortality rates of ACP induced by 1.0% LY reduced from 87% at 0.5 d to 37% at 2 d, and 0.5% LY treatments decreased from 73% to 20% during the same interval.

Exposure to light rain for 2 days with 8 mm cumulative rainfall significantly decreased the efficacy of CPF, THX, and PPF against ACP nymphs. The mortality rates of CPF, THX, and PPF were 13%, 57%, and 20% at 2 d, respectively. When mixed with LY at a rate of 0.2%, the three mixtures showed similar control efficacy with individual CPF, THX, and PPF throughout the experimental period, with no significantly enhanced efficacy. However, when mixed with LY at rates of 1.0% and 0.5%, the three insecticides effectively mitigated the rapid decrease in toxicity after light rain exposure, particularly 1.0% LY. The mortality rates of CPF&1.0%LY, THX&1.0%LY, and PPF&1.0%LY were 47%, 70%, and 43% at 2 d, respectively. Among the three mixtures, THX&1.0%LY maintained a mortality rate of 47% at 5 d (14 mm cumulative rainfall). When the cumulative rainfall reached 21 mm at 7 d, no statistically significant activity was detected in all treatments. These results demonstrate that the addition of 1.0% LY to insecticides, particularly THX, can significantly improve rainfastness and maintain higher efficacy against ACP under light rain conditions.

Table 4 shows that moderate rainfall significantly affected the efficacy of three insecticides (CPF, PPF, and THX) and mineral oil LY, both individually and in mixtures, against ACP nymphs. Within the first day after foliar application, a slight reduction in the insecticidal activity against ACP was observed in all treatments following exposure to light rainfall (about 2 mm) compared to observations during no rain days (Table 2). However, the efficacy of all treatments dramatically decreased when exposed to 24 mm of rainfall at 2 d post-application, with no significant insecticidal activity detected for any of the treatments.

### 3.3. THX Deposition on Citrus Leaves with LY Additions

**Figure 1:**
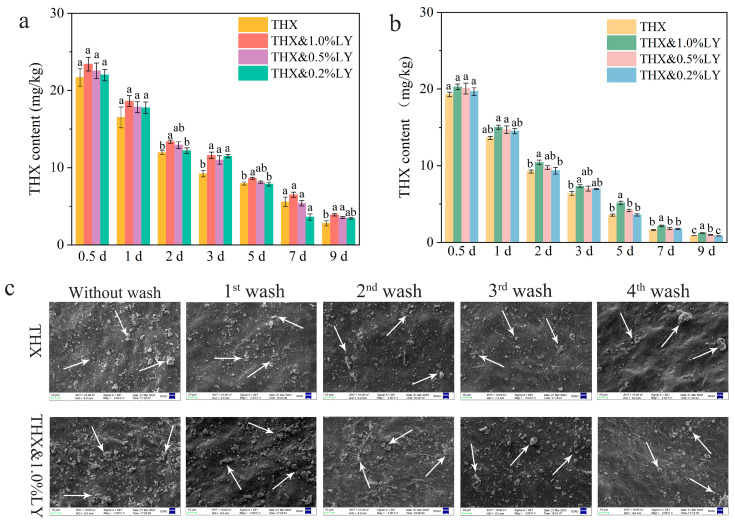
Changes in THX contents on citrus leaves at different times after spraying in the field experiment under (**a**) no rain conditions and under (**b**) light rain. Means with the same letters in the same time are not significantly different from each other (*p* > 0.05, LSD test). (**c**) SEM images of THX adhesion on citrus leaves before and after washing. Arrows represent THX particles.

To explore the relationship between insecticide toxicity and retention with LY additions, THX residues on citrus leaves were measured at different times after spraying. Based on previous results showing superior efficacy against ACP under different weather conditions, we focused on THX mixed with LY at 1.0%, 0.5%, and 0.2% (*v*/*v*) for this analysis. Under days without rainfall (Figure 1a), THX mixed with different concentrations of LY showed slightly higher retention on leaves compared to THX alone, although differences were not statistically significant at all time points. This trend was consistent across the observed period, suggesting that LY may enhance THX adherence to leaf surfaces. Under mid-rainfall conditions (Figure 1b), the mixture of THX and 1.0% LY resulted in significantly higher THX residue within leaves compared to other treatments.

SEM was employed to visualize the adhesion morphology of THX on citrus leaves with and without 1.0% LY addition (Figure 1c). The THX&1.0%LY mixture exhibited an increase in deposition of THX on the leaf surface compared to THX alone. Notably, this enhanced deposition persisted even after multiple rounds of simulated rainfall, providing strong evidence for LY’s ability to improve THX adhesion to leaf surfaces.

### 3.4. Wetting Behavior of THX with LY Additions

**Figure 2:**
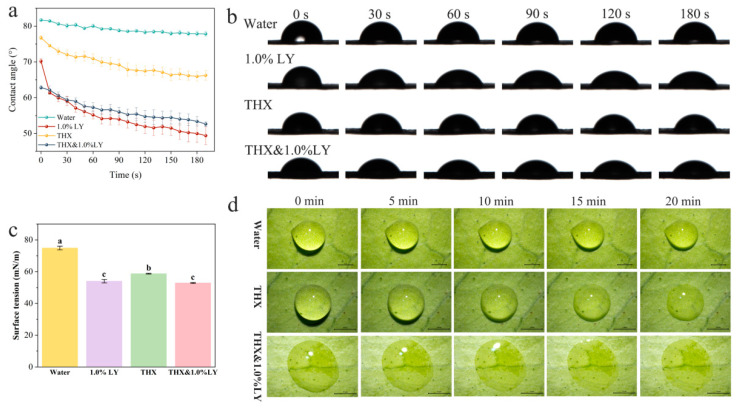
(**a**) Changes in droplet angles over time and (**b**) photographs of contact angles of droplets on citrus leaves with different treatments within 210 s. Means with the same letters are not significantly different from each other (*p* > 0.05, LSD test). (**c**) The surface tension of four liquids. (**d**) Spreading behavior of THX and THX&1.0%LY solutions on the surface of citrus leaves. Bars represent 1 mm.

Figure 2a,b shows the dynamic changes in contact angles for four liquids on citrus leaves. Compared to water, the contact angles for THX were lower and decreased from 76.76° to 66.24° within 210 s. The lowest contact angles were observed in 1.0% LY solution, and when LY was mixed with THX (THX&1.0%LY), a significant reduction in contact angles was achieved compared to individual THX. The surface tensions were measured as 74.90 mN/m for water, 54.05 mN/m for THX, 58.76 mN/m for 1.0% LY, and 52.87 mN/m for THX&1.0%LY (Figure 2c), which was positively correlated with the contact angles of the four liquids. The droplets of the THX&1.0%LY mixture diffused more rapidly on citrus leaves than individual THX on the leaves (Figure 2d).

### 3.5. LY Changed Mouthpart Morphology and Blocked Spiracle of ACP

**Figure 3:**
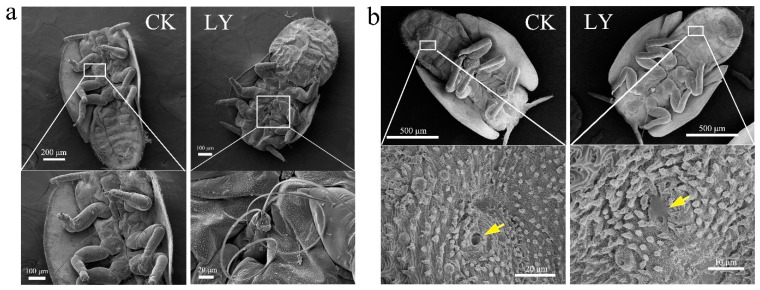
Effects of LY at 1.0% on mouthpart morphology (**a**) and spiracle (**b**). Arrows represent spiracle.

As shown in Figure 3a, in normal ACP nymphs, the stylet bundle, which likes a needle comprising a pair of mandibular and maxillary stylets and is located between the first and second pair of legs, was clearly observed. However, after treatment with LY, the stylet bundle was torn from its base. When ACP crawled across the leaves with LY solution on the surface, LY was embedded in or adhered to the spiracles of the cuticle (Figure 3b).

### 3.6. Discussion

Mixtures of pesticides with different modes of action can achieve equal toxicity to the total toxicity of its components, and sometimes exhibit much more toxic than the predicted amount [40], which contributes to delaying the evolved insecticide resistance and reducing the application volumes of pesticides. Mineral oils are a kind of petroleum-derived substance by means of a specific refinement. In agricultural pest management, most mineral oils with *n*C24 show negative effects on many insect pests [41,42]. In this study, LY at higher rates (1.0% and 0.5% *v*/*v*) exhibited strong insecticidal activity against ACP nymphs, with residue activity lasting 5–7 d under the no rain condition. In Florida, mineral oils are recommended at rates of 1–2% in 937 L ha^−1^ of water at citrus orchards, and Tansey et al. [33] suggested that high-frequency low-volume (every two weeks at 18.7 L ha^−1^) sprays significantly reduced ACP adult and nymph populations. Leong et al. [22] reported that mineral oil at 0.5% increased eggs, nymphs, and adult mortality under field conditions. Mineral oils are relatively harmless to human and beneficial insects [43], which can display synergistic activity [44]. Hence, mineral oils can serve as an insecticide or a surfactant, mixing with other insecticides to improve the control effects. The results of this study show that CPF, PPF, and THX, mixed with LY at a rate of 1.0%, significantly increased and prolonged protection for citrus trees against ACP during no rain days, while adding LY at 0.2% did not result in enhanced effects for CPF, PPF, and THX. The combined effects of a binary mixture of insecticides are influenced not only by their modes of action, but also by the specific ratios in which they are applied. For example, fluralaner mixed with chlorpyrifos at a mass ratio of 3:2 showed a strong synergistic effect against adult females of *Laodelphax striatellus*, whereas mass ratios of 4:1, 1:4, and 2:3 exhibited additive effects [45]. Mixtures of beta-cyfluthrin and thiamethoxam at mass ratios of 7:15, 9:13, 13:9, and 15:7 had synergistic effects against ACP adults, while a 11:11 mass ratio had an additive effect [26].

In the field, applications of pesticides are commonly limited by weather conditions such as rainfall. Most pesticides are prone to be washed off of plant surfaces when precipitation occurs during or shortly after foliar application, which not only results in high risks for aquatic organism, but also significantly reduces their efficacy [38]. A pesticide’s ability to withstand rainfall is an important factor that affects the efficacy of pesticides applied via spraying; thus, improving the adhesion of pesticides to plants contributes to increasing their efficacy and utilization. In this study, mixtures of CPF, PPF, and THX mixed with higher rates of LY mitigated the rapid decrease in toxicity of the synthetic pesticides under light rainfall. However, when the cumulative rainfall exceeded 20 mm, all treatment mixtures showed no control efficacy. The results of THX residues and SEM observation suggest that LY mineral oil increased a slight deposition of THX and significantly enhanced THX’s rainfastness to light rainfall, allowing for potentially enhanced and prolonged efficacy. Comparing THX residues between no rain and rainy conditions revealed a more rapid loss or degradation of THX after rain exposure. This observation underscores the impact of rainfall on pesticide retention and highlights the potential benefits of using adjuvants like LY to mitigate weather-related losses. The wetting and spreading behavior of droplets on plant leaf surfaces affected the deposition of solution on the surface [46]. He et al. [47] showed that nonionic surfactants markedly reduced the contact angle of Hexa droplets, which improved the pesticide wetting on the surfaces of cucumber powdery mildew leaves. Li et al. [48] indicated that the retention of glyphosate solution on different types of weeds increased with higher concentrations of alkyl polyglucoside (APG), which lowered surface tension between glyphosate droplets and weed leaf surfaces, and APG additions improved the control efficacy of glyphosate against weeds. The results of our work demonstrate that the THX solution had better wetting behavior on citrus leaf surfaces when 1.0% LY was added, which reduced surface tension and contact angles between the droplets and leaf surfaces, thus enhancing deposition of THX on leaves. Furthermore, adjuvants promote the adhesion force of compounds on plant leaves [49]. Hence, a slight increase in rainfastness in mixtures of THX and LY was found in this study.

However, the increased THX content was not equal to increased toxicity, which may be due to the insecticidal activity of LY mineral oil against ACP. In this study, 1.0% and 0.5% of LY showed excellent efficacy against ACP nymphs under no rain and light rain conditions. Buteler and Stadler [50] reported that mineral oils exhibited contact activity by disrupting respiration, impairing membrane function, and affecting feeding behavior. Our previous study indicated that mineral oils inhibited feeding of *D. citri* by causing them to raise their heads and upper abdomens to form a half-erect posture [31]. ACP acquires and transmits HLB pathogens by feeding. ACP has a longer piercing–sucking mouthpart that includes a stylet bundle like a needle composed of a pair of mandibular and maxillary stylets [51]. The two maxillary stylets form the food and salivary canals and the mandibular stylets are positioned on each lateral side of the maxillary stylets, which helps the maxillary stylets penetrate plant tissues [51,52]. In the present study, SEM observations found that LY mineral oil caused the stylet bundle to tear from the base, which influenced the stylet penetration and subsequently reduced feeding behavior. On the other hand, we also observed that mineral oil was embedded in or adhered to the spiracles of the cuticle. This finding is consistent with numerous studies which show that the mode of action of mineral oils is to block spiracle, leading to insect suffocation [50].

## Figures and Tables

**Table 1:** The weather conditions following each application.

Application Date	Weather Conditions	Daily Rainfall							
		0.5 Day	1 Day	2 Days	3 Days	4 Days	5 Days	7 Days	9 Days
April 24	No rainfall	0 mm	0 mm	0 mm	0 mm	0 mm	0 mm	0 mm	0 mm
April 3	Light rain *	0 mm	5 mm	3 mm	2 mm	3 mm	3 mm	7 mm	4 mm
June 10	Moderate rain *	0 mm	2 mm	22 mm	NA	NA	NA	NA	NA

* Rain intensity is classified according to Grade of Precipitation (GB/T 28592-2012) [39]. NA: Not available.

**Table 2:** Mortality rates of ACP nymphs caused by the insecticides mixed with LY under no rain days.

Treatment Group	Treatment	Mean Mortality Rate (±SE)%						
		0.5 Day	1 Day	2 Days	3 Days	5 Days	7 Days	9 Days
LY	1% LY	83 ± 9 ab	87 ± 9 b	93 ± 7 ab	73 ± 7 c	67 ± 3 c	27 ± 3 bc	3 ± 3 b
	0.5% LY	90 ± 6 ab	63 ± 9 b	53 ± 7 c	37 ± 3 d	20 ± 6 f	3 ± 3 cd	0 ± 0 b
	0.2% LY	30 ± 6 c	10 ± 6 d	13 ± 3 e	0 ± 0 e	0 ± 0 g	0 ± 0 d	0 ± 0 b
CPF	CPF	83 ± 9 ab	50 ± 10 c	57 ± 3 c	47 ± 7 d	47 ± 3 d	3 ± 3 cd	0 ± 0 b
	CPF&1.0%LY	100 ± 0 a	93 ± 7 ab	87 ± 7 ab	80 ± 6 bc	83 ± 3 b	63 ± 3 a	0 ± 0 b
	CPF&0.5%LY	97 ± 3 ab	73 ± 7 b	73 ± 7 b	63 ± 3 c	67 ± 7 c	17 ± 3 c	0 ± 0 b
	CPF&0.2%LY	87 ± 7 ab	53 ± 7 c	50 ± 6 c	43 ± 3 d	33 ± 3 e	10 ± 3 cd	0 ± 0 b
THX	THX	93 ± 7 ab	100 ± 0 a	100 ± 0 a	97 ± 3 ab	87 ± 3 ab	37 ± 3 b	3 ± 3 b
	THX&1.0%LY	100 ± 0 a	100 ± 0 a	100 ± 0 a	100 ± 0 a	97 ± 3 a	73 ± 3 a	10 ± 6 a
	THX&0.5%LY	100 ± 0 a	100 ± 0 a	100 ± 0 a	93 ± 3 ab	93 ± 3 ab	73 ± 3 a	0 ± 0 b
	THX&0.2%LY	100 ± 0 a	100 ± 0 a	83 ± 3 b	83 ± 3 bc	87 ± 3 ab	63 ± 9 a	0 ± 0 b
PPF	PPF	87 ± 3 ab	57 ± 3 c	47 ± 7 cd	40 ± 6 d	20 ± 0 f	10 ± 6 cd	0 ± 0 b
	PPF&1.0%LY	87 ± 7 ab	100 ± 0 a	83 ± 9 b	87 ± 3 b	77 ± 3 bc	33 ± 3 b	0 ± 0 b
	PPF&0.5%LY	80 ± 12 b	90 ± 6 ab	70 ± 6 bc	67 ± 7 c	60 ± 6 c	10 ± 6 cd	0 ± 0 b
	PPF&0.2%LY	87 ± 9 ab	40 ± 6 d	33 ± 7 d	37 ± 3 d	30 ± 6 ef	3 ± 3 cd	0 ± 0 b
CK	Water	0 ± 0 d	0 ± 0 e	0 ± 0 e	0 ± 0 e	0 ± 0 g	0 ± 0 d	0 ± 0 b

Means with the same letters in the same column are not significantly different from each other (*p* > 0.05, LSD test).

**Table 3:** Effects of light rainfall on the control efficacy of the insecticides mixed with LY against ACP nymphs.

Treatment Group	Treatment	Mean Mortality Rate (±SE)%						
		0.5 Day	1 Day	2 Days	3 Days	5 Days	7 Days	9 Days
LY	1% LY	87 ± 7 b	73 ± 3 bc	37 ± 3 c	10 ± 6 d	10 ± 6 cd	0 ± 0 c	0 ± 0 a
	0.5% LY	73 ± 3 c	57 ± 3 cd	20 ± 6 d	3 ± 3 d	3 ± 3 d	0 ± 0 c	0 ± 0 a
	0.2% LY	10 ± 6 d	3 ± 3 e	0 ± 0 e	0 ± 0 d	0 ± 0 d	0 ± 0 c	0 ± 0 a
CPF	CPF	73 ± 3 c	33 ± 3 d	13 ± 6 d	3 ± 3 d	3 ± 3 d	0 ± 0 c	0 ± 0 a
	CPF&1.0%LY	97 ± 3 ab	87 ± 3 ab	47 ± 3 bc	20 ± 6 cd	13 ± 3 c	3 ± 3 bc	0 ± 0 a
	CPF&0.5%LY	93 ± 3 ab	53 ± 9 cd	20 ± 6 d	17 ± 3 cd	7 ± 3 cd	3 ± 3 bc	0 ± 0 a
	CPF&0.2%LY	83 ± 3 bc	43 ± 9 d	13 ± 3 d	7 ± 3 d	3 ± 3 d	0 ± 0 c	0 ± 0 a
THX	THX	100 ± 0 a	83 ± 3 b	57 ± 7 b	37 ± 3 b	27 ± 3 b	7 ± 3 b	0 ± 0 a
	THX&1.0%LY	100 ± 0 a	100 ± 0 a	70 ± 6 a	53 ± 3 a	47 ± 3 a	17 ± 3 a	3 ± 3 a
	THX&0.5%LY	100 ± 0 a	100 ± 0 a	60 ± 6 ab	37 ± 3 b	27 ± 3 b	13 ± 3 a	3 ± 3 a
	THX&0.2%LY	100 ± 0 a	97 ± 3 ab	57 ± 7 b	20 ± 6 cd	13 ± 3 c	3 ± 3 bc	0 ± 0 a
PPF	PPF	70 ± 6 c	43 ± 7 d	20 ± 6 d	3 ± 3 d	3 ± 3 d	0 ± 0 c	0 ± 0 a
	PPF&1.0%LY	97 ± 3 ab	67 ± 3 c	43 ± 3 c	23 ± 3 c	10 ± 6 cd	3 ± 3 bc	0 ± 0 a
	PPF&0.5%LY	90 ± 6 ab	53 ± 9 cd	17 ± 3 d	17 ± 3 cd	3 ± 3 d	0 ± 0 c	0 ± 0 a
	PPF&0.2%LY	77 ± 3 bc	57 ± 3 cd	17 ± 3 d	0 ± 0 d	0 ± 0 d	0 ± 0 c	0 ± 0 a
CK	Water	0 ± 0 d	0 ± 0 e	0 ± 0 e	0 ± 0 d	0 ± 0 d	0 ± 0 c	0 ± 0 a

Means with the same letters in the same column are not significantly different from each other (*p* > 0.05, LSD test.

**Table 4:** Effects of moderate rainfall on the control efficacy of the insecticides mixed with LY against ACP nymphs.

Treatment Group	Treatment	Mean Mortality Rate (±SE)%	
		1 Day	2 Days
LY	1% LY	77 ± 3 ab	3 ± 3 ab
	0.5% LY	60 ± 6 bc	3 ± 3 ab
	0.2% LY	13 ± 3 e	0 ± 0 b
CPF	CPF	47 ± 3 c	10 ± 3 a
	CPF&1.0%LY	83 ± 3 ab	3 ± 3 ab
	CPF&0.5%LY	53 ± 9 c	3 ± 3 ab
	CPF&0.2%LY	30 ± 6 d	0 ± 0 b
THX	THX	73 ± 3 ab	3 ± 3 ab
	THX&1.0%LY	87 ± 3 a	3 ± 3 ab
	THX&0.5%LY	80 ± 6 ab	0 ± 0 b
	THX&0.2%LY	77 ± 9 ab	3 ± 3 ab
PPF	PPF	40 ± 6 cd	3 ± 3 ab
	PPF&1.0%LY	70 ± 6 b	3 ± 3 ab
	PPF&0.5%LY	53 ± 9 c	0 ± 0 b
	PPF&0.2%LY	47 ± 3 c	0 ± 0 b
CK	Water	0 ± 0 e	0 ± 0 b

Means with the same letters in the same column are not significantly different from each other (*p* > 0.05, LSD test).

## Data Availability

Data are contained within the article.

## References

[1] Wu J., Cao J.P., Chen J.B., Huang L.X., Wang Y., Sun C., Sun C.D. (2023). Detection and classification of volatile compounds emitted by three fungi-infected citrus fruit using gas chromatography-mass spectrometry. Food Chem..

[2] Gao A., Tian Z.W., Ma W., Song Y.P., Ren L.L., Feng Y.L., Qian J.P., Xu L.J. (2024). Fruits hidden by green: An improved YOLOV8n for detection of young citrus in lush citrus trees. Front. Plant Sci..

[3] Li Z.X., Zhang Y.H., Zhao Q.Y., Wang C.Q., Cui Y.L., Li J., Chen A.H., Liang G.L., Jiao B.N. (2020). Occurrence, temporal variation, quality and safety assessment of pesticide residues on citrus fruits in China. Chemosphere.

[4] Bové J.M. (2006). Huanglongbing: A destructive, newly-emerging, century-old disease of citrus. J. Plant Pathol..

[5] Roldan E.L., Stelinski L.L., Pelz-Stelinski K.S. (2023). Foliar antibiotic treatment reduces *Candidatus liberibacter* Asiaticus acquisition by the Asian citrus psyllid, *Diaphorina citri* (Hemiptera: Liviidae), but does not reduce tree infection rate. J. Econ. Entomol..

[6] Halbert S.E. (2005). The discovery of huanglongbing in Florida. Proceedings of the 2nd International Citrus Canker and Huanglongbing Research Workshop.

[7] Hu W., Zhao C.F., Zheng R.K., Duan S., Lu Z.J., Zhang Z.X., Yi L., Zhang N. (2024). *Serratia marcescens* induces apoptosis in *Diaphorina citri* gut cells via reactive oxygen species-mediated oxidative stress. Pest Manag. Sci..

[8] Endarto O., Wicaksono R.C., Wuryantini S., Tarno H., Supoyo N. (2024). Climate change mitigation and seasonal infestation patterns of citrus psyllid *Diaphorina citri*: Implications for managing huanglongbing (HLB) disease in tangerine citrus. IOP Conf. Ser. Earth Environ. Sci..

[9] Narouei-Khandan Hossein A., Halbert S.E., Worner S.P., van Bruggen A.H.C. (2016). Global climate suitability of citrus huanglongbing and its vector, the Asian citrus psyllid, using two correlative species distribution modeling approaches, with emphasis on the USA. Eur. J. Plant Pathol..

[10] Inoue H., Ohnishi J., Ito T., Tomimura K., Miyata S., Iwanami T., Ashihara W. (2009). Enhanced proliferation and efficient transmission of *Candidatus liberibacter* Asiaticus by adult *Diaphorina citri* after acquisition feeding in the nymphal stage. Ann. Appl. Biol..

[11] Chen X.D., Ashfaq M., Stelinski L.L. (2018). Susceptibility of Asian citrus psyllid, *Diaphorina citri* (Hemiptera: Liviidae), to the insecticide afidopyropen: A new and potent modulator of insect transient receptor potential channels. Appl. Entomol. Zoolog..

[12] Chen X.D., Seo M., Stelinski L.L. (2017). Behavioral and hormetic effects of the butenolide insecticide, flupyradifurone, on Asian citrus psyllid, *Diaphorina citri*. Crop Prot..

[13] Qureshi J.A., Kostyk B.C., Stansly P.A. (2014). Insecticidal suppression of Asian citrus psyllid *Diaphorina citri* (Hemiptera: Liviidae) vector of huanglongbing pathogens. PLoS ONE.

[14] De Carli L.F., Miranda M.P., Volpe H.X.L., Zanardi O.Z., Vizoni M.C., Martini F.M., Lopes J.P.A. (2018). Leaf age affects the efficacy of insecticides to control Asian citrus psyllid, *Diaphorina citri* (Hemiptera: Liviidae). J. Appl. Entomol..

[15] Farmanullah, Badshah H., Gul R. (2005). Evaluation of six different groups of insecticides for the control of citrus psylla *Diaphorina citri* (Hemiptera: Psyllidae). Wārasān Songkhlā Nakharin.

[16] Nauen R., Ebbinghaus-Kintscher U., Salgado V.L., Kaussmann M. (2003). Thiamethoxam is a neonicotinoid precursor converted to clothianidin in insects and plants. Pestic. Biochem. Physiol..

[17] Hu W., Kuang F., Chun J., Lu Z.J., Li X.T., Zhao Q.Y., Zhong B.L., Su H.N., Zhang Z.X., Zhang N. (2019). Uptake of soil-applied thiamethoxam in orange and its effect against Asian citrus psyllid in different seasons. Pest Manag. Sci..

[18] Tang T., Zhao M.P., Wang P., Huang S.K., Fu W. (2021). Control efficacy and joint toxicity of thiamethoxam mixed with spirotetramat against the Asian citrus psyllid, *Diaphorina citri* Kuwayama. Pest Manag. Sci..

[19] Byrne F.J., Daugherty M.P., Grafton-Cardwell E.E., Bethke J.A., Morse J.G. (2017). Evaluation of systemic neonicotinoid insecticides for the management of the Asian citrus psyllid *Diaphorina citri* on containerized citrus. Pest Manag. Sci..

[20] Boina D.R., Rogers M.E., Wang N., Stelinski L.L. (2010). Effect of pyriproxyfen, a juvenile hormone mimic, on egg hatch, nymph development, adult emergence and reproduction of the Asian citrus psyllid, *Diaphorina citri* Kuwayama. Pest Manag. Sci..

[21] Tiwari S., Clayson P.J., Kuhns E.H., Stelinski L.L. (2012). Effects of buprofezin and diflubenzuron on various developmental stages of Asian citrus psyllid, *Diaphorina citri*. Pest Manag. Sci..

[22] Leong S.S., Leong S.C.T., Beattie G.A.C. (2021). Effect of horticultural mineral oil on huanglongbing transmission by *Diaphorina citri* Kuwayama (Hemiptera: Psyllidae) population in a commercial citrus orchard in Sarawak, Malaysia, Northern Borneo. Insects.

[23] Tiwari S., Gondhalekar A.D., Mann R.S., Scharf M.E., Stelinski L.L. (2011). Characterization of five cyp4 genes from asian citrus psyllid and their expression levels in *Candidatus liberibacter* Asiaticus-infected and uninfected psyllids. Insect Mol. Biol..

[24] Hu H.Q., Wang X.D., Lin X.J., Yang C.Y., Chen J., Fan G.C. (2020). Field efficacy of spirotetramat·bifenthrin 26% SC against *Diaphorina citri* Kuwayama. Agrochemicals.

[25] Song X.B., Peng A.T., Chen X., Cheng B.P., Zhou J., Ling J.F. (2015). Control effects of six pesticides such as beta-cypermethrin·thiamethoxam on citrus psyllid. Agrochemicals.

[26] Tang T., Zhao M., Wang P., Xiao Y., Huang S., Fu W. (2020). Field efficacies and joint actions of beta-cyfluthrin mixed with thiamethoxam or tolfenpyrad against *Diaphorina citri* (Hemiptera: Liviidae). J. Econ. Entomol..

[27] Singh H., Cheema H.K., Singh R. (2020). Field evaluation of horticultural mineral oils and botanicals against bean thrips, *Megalurothrips distalis* (Karny) (Thysanoptera: Thripidae), in summer mung bean. Egypt. J. Biol. Pest Control.

[28] Martín López B., Varela I., Marnotes S., Cabaleiro C. (2006). Use of oils combined with low doses of insecticide for the control of *Myzus persicae* and PVY epidemics. Pest Manag. Sci..

[29] Rae D.J., Beattie G.A.C., Watson D.M., Liu Z.M., Jiang L. (1996). Effects of petroleum spray oils without and with copper fungicides on the control of citrus leafminer, *Phyllocnistis citrella* Stainton (Lepidoptera: Gracillariidae). Aust. J. Entomol..

[30] Jeppson L.R., Carman G.E. (1974). Low volume applications to citrus trees: Effectiveness in control of citrus red mite and California red scale with petroleum oils and pesticides. J. Econ. Entomol..

[31] Hu W., Zheng R.K., Feng X.L., Kuang F., Chun J., Xu H.H., Chen T.T., Lu J.H., Li W.M., Zhang N. (2023). Emergence inhibition, repellent activity and antifeedant responds of mineral oils against Asian citrus psyllid, *Diaphorina citri* (Hemiptera: Liviidae). Int. J. Pest Manag..

[32] Ouyang G.C., Fang X.D., Lu H.L., Zhou X., Meng X., Yu S.K., Guo M.F., Xia Y.L. (2013). Repellency of five mineral oils against *Diaphorina citri* (Hemiptera: Liviidae). Fla. Entomol..

[33] Tansey J.A., Jones M.M., Vanaclocha P., Robertson J., Stansly P.A. (2015). Costs and benefits of frequent low-volume applications of horticultural mineral oil for management of Asian citrus psyllid, *Diaphorina citri* Kuwayama (Hemiptera: Psyllidae). Crop Prot..

[34] Rae D., Liang W., Watson D., Beattie G. (1997). Evaluation of petroleum spray oils for control of the Asian citrus psylla, *Diaphorina citri* (Kuwayama) (Hemiptera: Psyllidae), in China. Int. J. Pest Manag..

[35] Malik K., Raghavendra K.V., Kumar M. (2020). Evaluation of thiacloprid and mineral oil combination against sucking pests of potato. Indian J. Entomol..

[36] Boina D.R., Bloomquist J.R. (2015). Chemical control of the Asian citrus psyllid and of huanglongbing disease in citrus. Pest Manag. Sci..

[37] Boina D., Onagbola E., Salyani M., Stelinski L. (2009). Influence of post treatment temperature on the toxicity of insecticides against *Diaphorina citri* (Hemiptera: Psyllidae). J. Econ. Entomol..

[38] Martínez-Megías C., Mentzel S., Fuentes-Edfuf Y., Moe S.J., Rico A. (2023). Influence of climate change and pesticide use practices on the ecological risks of pesticides in a protected mediterranean wetland: A bayesian network approach. Sci. Total Environ..

[39] (2012). Grade of Precipitation.

[40] Olfati Somar R., Zamani A.A., Alizadeh M. (2019). Joint action toxicity of imidacloprid and pymetrozine on the melon aphid, *Aphis gossypii*. Crop Prot..

[41] Lai D.T., Khuc H.D., Nguyen L.V., Hong K., Nguyen H.N. (2022). Potential of using mineral oils for the control of the mosquito bugs *Helopeltis theivora* (Hemiptera: Miridae) in cashew plantations. J. Asia-Pac. Entomol..

[42] Singh S., Protasov A., Kramer R.M., Yaacobi G., Kaspi R. (2023). Toxicity assessment of common acaricides and mineral oils on Anagyrus vladimiri, an effective biocontrol agent of citrus mealybug. J. Econ. Entomol..

[43] Leong S.C.T., Abang F., Beattie A., Kueh R.J.H., Wong S.K. (2012). Impacts of morticultural mineral oils and two insecticide practices on population fluctuation of *Diaphorina citri* and spread of huanglongbing in a citrus orchard in Sarawak. Sci. World J..

[44] Kimber I., Carrillo J.C. (2016). Oral exposure to mineral oils: Is there an association with immune perturbation and autoimmunity?. Toxicology.

[45] Ling H., Li C., Tang T., Liu D., Zhao C.Q. (2019). Activity of fluralaner with three insecticides and joint action of their mixtures against *Laodelphax striatellus*. J. Environ. Entomol..

[46] He L.F., Ding L., Zhang P., Li B.X., Mu W., Liu F. (2021). Impact of the equilibrium relationship between deposition and wettability behavior on the high-efficiency utilization of pesticides. Pest Manag. Sci..

[47] He L.F., Xi S.W., Ding L., Li B.X., Mu W., Li P.Q., Liu F. (2022). Regulating the entire journey of pesticide application on surfaces of hydrophobic leaves modified by pathogens at different growth stages. Acs Nano.

[48] Li Z.L., Zhang X.Y., Wang Y., Zheng Z.R., Zhang C.H., Wu T.Y., Wu Y.L., Gao Y.X., Du F.P. (2023). Improved method to characterize leaf surfaces, guide adjuvant selection, and improve glyphosate efficacy. J. Agric. Food Chem..

[49] Bhushan B., Jung Y.C. (2008). Wetting, adhesion and friction of superhydrophobic and hydrophilic leaves and fabricated micro/nanopatterned surfaces. J. Phys. Condens. Matter.

[50] Buteler M., Stadler T. (2011). A Review on the Mode of Action and Current Use of Petroleum Distilled Spray Oils.

[51] Garzo E., Bonani J.P., Lopes J.R.S., Fereres A. (2012). Morphological description of the mouthparts of the Asian citrus psyllid, *Diaphorina citri* Kuwayama (Hemiptera: Psyllidae). Arthropod Struct. Dev..

[52] Zhao L., Dai W., Zhang C., Zhang Y. (2010). Morphological characterization of the mouthparts of the vector leafhopper *Psammotettix striatus* (L.) (Hemiptera: Cicadellidae). Micron.

